# Critical Success Factors of a Drug Traceability System for Creating Value in a Pharmaceutical Supply Chain (PSC)

**DOI:** 10.3390/ijerph16111972

**Published:** 2019-06-04

**Authors:** Ronaldo Brito da Silva, Claudia Aparecida de Mattos

**Affiliations:** Production Engineering Department, Centro Universitário FEI, 09850-901 Sao Bernardo, Brazil; ronaldobrito71@hotmail.com

**Keywords:** traceability, value creation, critical success factors (CSFs), drugs, analytic hierarchy process (AHP), pharmaceutical supply chain (PSC)

## Abstract

The general objective of this study was to identify and prioritize the critical success factors required for the adoption of a system to create value for pharmaceutical supply chain stakeholders, and the pharmaceutical supply network as a whole, by using a multi-perspective framework that combines elements of the technology–organization–environment (TOE) contexts for enterprises. The methodology is based on a literature review and expert interviews following the analytic hierarchy process (AHP). This paper identifies and prioritizes 18 critical success factors from three categories: technological, organizational, and environmental. From a practical point of view, this research contributes to the literature by providing expert insight on the topic of drug traceability, especially in terms of how possible values can be captured by companies.

## 1. Introduction

The development of advanced information technology (IT) solutions has enabled the formation of inter-organizational networks which facilitate the processing and sharing of information in real time, thereby allowing several controls, including traceability in supply chains [1,2]. As the manufacture, supply, and distribution of drugs becomes more complex, there is an increased need for innovative technology-based solutions to protect patients globally via traceability systems [3]. Traceability has become a differentiator in many industries. The pharmaceutical industry, along with the food industry, has been capturing the interest of researchers more than other industries, due to its more stringent regulation by governments and international organizations, while safety and quality in the industry have caught the world’s attention [4]. However, the implementation of effective drug traceability systems faces many challenges, and it is essential to identify the critical success factors (CSFs) for such systems. There are theoretical gaps in the study of the development of traceability in different pharmaceutical supply chains, such as the creation of value by the implementation of traceability systems, as discussed by [5,6]. In references [5,7], the authors argue that the primary reason for adopting traceability may be to respond to legal claims, and that companies that use new technologies do not make the most of the potential operational benefits of traceability. For these reasons, it is necessary to understand the potential values which can be obtained with traceability, and to understand the CSFs to reach these values. It is critical to identify CSFs in order to ensure the effective implementation of a traceability system and to leverage improvements in its performance [8]. The lack of further discussion of the values of a traceability system in the drug supply chain, and the difficulty of the implementation and diffusion of such systems, indicate that more studies on drug traceability are needed. Based on this research gap, the following questions were formulated to guide this research:

(1) What are the CSFs for the implementation of a drug traceability system in the pharmaceutical supply chain (PSC)?
(2) What is the relative importance of each CSF for the implementation of a traceability system in the drug supply chain?

Based on these questions, the overall objective of this study was to identify and prioritize the CSFs for the adoption of a drug traceability system to create value for PSC stakeholders and the supply network as a whole. For the development of this research, we initially identified CSFs in the literature, and secondly analyzed and prioritized these CSFs using the analytic hierarchy process (AHP) method, based on interviews with experts involved in the PSC in Brazil. The development of a drug traceability system is of great importance for Brazil, since counterfeit medicine accounts for 19% of the medicine sold in the country [World Health Organization]. Far from being an exclusively Brazilian problem, the same WHO report points out that counterfeit drugs account for 10% of the medicine sold globally, while in some places in South America the average value can reach 30%. 

## 2. Pharmaceutical Supply Chain

The pharmaceutical sector currently accounts for 8% of Brazil’s Gross Domestic Product (GDP), contributing 75,000 direct and 500,000 indirect jobs [9]. By April 2022, all Brazilian drugs will have to comply with the regulations set by the National Agency of Sanitary Vigilance (ANVISA), which enable drug traceability. ANVISA, a government agency, is considering a model in which the agency centralizes drug traceability, similar to models which have been adopted in countries recognized for their drug traceability models, such as Turkey and Argentina.

The value of the global pharmaceutical market is expected to reach around US $ 1.4 trillion by 2020; the value of Brazil’s pharmaceutical market currently ranks seventh in the world, and is expected to rise to fifth by 2020 [10].

In reference [11], the author discusses processes, operations, and organizations involved in the discovery, development, and manufacture of drugs and medications [12]. PSCs are socio-technical systems designed to align firms in order to enable improved health status through the provision of medicines. Complementary and alternative products and process technologies may coexist within such a system [13]. A PSC is a five-tier supply chain which includes [14]:

- Primary manufacturers, who are responsible for the production of required active ingredients (RAI)
- Secondary manufacturers, who are responsible for further production processes (manufacturing centers)
- Main distribution centers
- Local distribution centers
- Destination zones/demand points

Other authors, such as in [15], define PSCs as organizations acting in different buying and selling capacities, including:

- Manufacturers (sellers)
- Wholesalers (buyers and sellers)
- Hospitals (buyers)

The aforementioned authors [11,12,13,14,15] consider that a PSC is composed of layers, namely a supplier, manufacturer, wholesaler, and pharmacy or hospital. The understanding of the concept of a PSC and the respective actors is an important factor for several research areas, including traceability, inter-organizational systems implementation, anti-counterfeiting strategies, and best practices in the PSC around such sophisticated tools [16,17].

PSCs are affected by counterfeiting and theft issues, which not only compromise the profits and reputations of manufacturers, wholesalers, pharmacies, and hospitals, but also compromise public health and consumer safety [18,19]. In this context, traceability plays a central role, particularly in supply chains where multiple parties are involved and in which rigorous criteria must be fulfilled to lead to a successful outcome [20,21,22].

### 2.1. Traceability Concept

According to [23], traceability is a mechanism to ensure safety. Traceability is defined by *The British Standars Institution* as: 

“The ability to trace the history, application, or location of an object. The object is defined by this same standard as being any perceivable or conceivable thing. Objects can be material (i.e., an engine, a sheet of paper, a diamond), non-material (i.e., a conversion rate, a design plan) or imagined (i.e., the future state of the organization)”.

When considering a product or service, traceability can be related to:

- The origin of materials and parts;
- Processing history;
- The distribution and location of a product or service after delivery.

In reference [24], traceability systems are described as systems designed to track the flow of products or product attributes through the production process or supply chain. In this context, digital technologies are transforming supply chains by allowing flexible production and automation and the use of sensors to track the location, quality, and authenticity of products.

In general terms, traceability requirements vary depending on the context. The information that needs to be collected is established based on the underlying needs and objectives of each organization. Enabling traceability is the most effective action for combating illegal trade, and for dealing with smuggling in general. The adherence of “the pharmaceutical industry to the use of traceability systems is a complex and often costly task that occurs in two ways: (1) voluntarily, when a company sees a competitive differential; and (2) obligatorily, through the imposition of standards and regulations [25,26]. Governmental regulations have increased the security of the distribution of pharmaceutical products. Regulatory platforms and their mandatory requirements are being adopted in several countries around the world, adding certain operational complexities to multinational manufacturing and distribution and forcing manufacturers and distributors to create flexible systems to serialize products shipped to different markets according to local regulatory requirements [4]. 

In recent decades, health-related information, such as electronic health records, and traceability, have brought new challenges for the management, storage, and processing of data. However, with the aid of technologies such as Cloud Computing and Big Data, health information in cyber-physical systems (CPS) can be more efficiently managed [27].

#### Description of Drug Traceability System

As discussed by Alonso-Rorís et al. (2016) [28], an important characteristic of a drug traceability system is the identification of the entities involved in the processes being studied.

**Figure 1:**
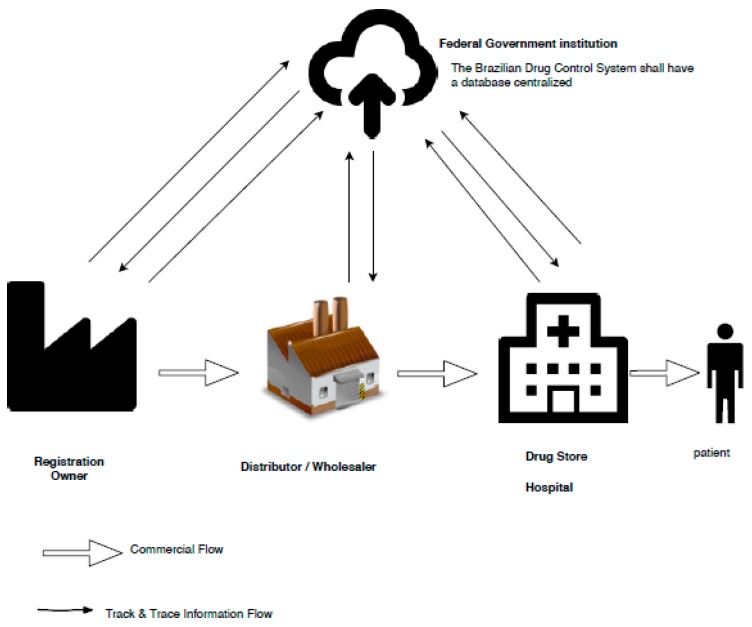
Traceability of medications in Brazil under the National Agency of Sanitary Vigilance (ANVISA) standard.

By April 2022, all Brazilian medications will have to meet the ANVISA standard which establishes the traceability of medications. Each player within the drug supply chain will have to report when a product passes through their facility. This has previously been the case, however, until now, pharmacies, distributors, and other trading partners fed supply chain information back to the marketing authorization holder (MAH). In contrast, after April 2022, information will be reported to a centralized reporting authority established by ANVISA. This centralized system will store and better analyze all information related to pharmaceutical products covered by the new law as presented in Figure 1.

The architecture of the ANVISA drug-traceability system is based on the automation pyramid model defined by the International Society of Automation (ISA). The reference technology architecture to support the National Drug Control System (NDS) was divided into five layers:

1. Government: a government central bank emulator and governmental services for receiving and sending data. This database or repository has the function of receiving data from all participants of the supply chain and storing it for future reference, allowing, for instance, the creation of an anomaly detection application;
2. Validation and Collaboration: in this layer, validation and collaboration services are used among the participants. This layer is intended to serve as a filter for the governmental layer, which has specific interests in receiving traceability events. This layer is also intended to share information among participants, allowing for collaboration by sharing information such as items sent from the drug registry holder to the hospital, which are consolidated on pallets, boarding boxes, and unit boxes;
3. Corporative: Corporative systems responsible for the corporative management of business processes (Enterprise Resources Planning (ERP) and support systems). The activities performed in this layer refer to the internal administrative procedures of each participant, such as the activities of production management, inventory, shipping, and receiving;
4. Supervisory systems: this layer contains the supervisory systems and interfaces and coordinates activities such as transfers, receipts, and internal distributions;
5. Operations: these layers are directly connected to operations involving the handling of drugs.

### 2.2. Critical Success Factors of the Drug Traceability System

Critical success factors (CSFs) are not detailed practices for the implementation of a system; rather, they are factors that support the highly successful implementation of systems and help the organization to focus on the most important factors [29,30]. The implementation of an effective traceability system faces many challenges, and it is essential to identify its CSFs [31]. The implementation of a traceability system requires an appropriate strategy, and the CSFs of systems that have been effectively implemented, and interaction among them, may help to develop an effective strategy for the systems [32]. Additionally, the implementation of traceability systems can be seen as the adoption of information systems in enterprises operating in a complex PSC. Thus, models of technology adoption as technology–organization–environment (TOE) provided useful guidance for developing this study [31]. IT developers should obtain a better understanding of non-technological factors in order to better plan their implementation. The TOE framework explains that three different contexts influence the decisions to adopt new technologies, namely technological context, organizational context, and environment. The TOE framework, as originally presented and later adapted in IT adoption studies, provides a useful analytical framework that can be used to study the adoption and assimilation of different types of IT innovations [31].

Ngai et al. (2007) [33] discussed critical factors regarding the use of Radio Frequency Identification (RFID) technology in the aeronautical industry, which they categorized into four categories: (1) raised organizational motivation for improvement; (2) implementation process; (3) cost control; and (4) university–industry interaction. 

Aung and Chang (2014) [34] mention that an efficient traceability system must be characterized by the amplitude (that is, the amount of collected information), the depth (the capacity to track relevant information), and the precision of information, thus allowing the balance of costs and benefits. Duan et al. (2017) [31] established a CSF framework for the implementation of traceability systems based on empirical evidence collected in China.

References [8,31] highlight the following dimensions of critical factors for the implementation of a traceability system:

- Environment (legislation, government support, standards);
- Organizational (involvement of top management, supplier support, effective communication);
- Technology (the quality of the tracked information and the traceability system).

Traceability is essentially a subsystem of quality management. The development of an advanced internal traceability system can also be stimulated by the attempt to improve and increase the efficiency of data collection, plant control, and quality assurance. Additionally, Moe (1998) states that there is a need to create a data model for tracking the variation in the quantity of unit-traceable resources at all times, or tracking the history of process activity [35]. 

Traceability systems require the sharing of information and the use of a standardized language, since data flow occurs between different companies. The implementation of a traceability system primarily focuses on rethinking the tasks and objectives of the entire management of the supply chain [36]. 

Rotunno et al. (2014) [4] argue that a traceability system for the pharmaceutical industry places additional demands on the architecture of the applications and components of the existing IT architecture of companies. This raises critical factors, such as the integration between exterior systems (horizontal integration) and the presence of a multi-layer architecture (vertical integration). Canavari et al. (2010) [37] mention internal factors (related to the mission of companies, structure, technology, and removal of limitations), the type of operators involved and their links, as well as the characteristics of the macroenvironment (legislation and competitive environment) and the influence of the internal factors on the choice of the information process. Due to the constraints and factors mentioned above, every company or supply chain has to define which strategies drive its activity and what kind of good or service it will provide to the market [37]. Based on the studies discussed in this article we consolidate the CSFs for the implementation of a traceability system (Table 1).

#### 2.2.1. The Success of the Drug Traceability System—Value Creation

Simchi-Levi and Fine (2010) [38] suggest that modern supply chains must be seen as value chains or networks of interconnected organizations in a win-win relationship with increasing interdependence. The implementation of a traceability system implies the development of a network; for it to be effective, it is imperative to create value for the actors and for the network as a whole. 

Lovis (2008) [39] affirmed that many potential benefits can be expected from a traceability system for the health sector, such as:

- An increase in the efficiency of the supply chain;
- The prevention of errors;
- The protection of public health (to prevent fraud and counterfeit products);
- Medical-legal investigations (the identification of actors and locations on the timeline);
- Clinical research and epidemiological surveillance (data source to leverage the use of clinical data);
- The management of flows and processes;
- Revenues (in the sense of facilitating controls);
- Fraud prevention measures;

The values that can be generated by a traceability system have been studied by several authors, such as [37], who present the impact on an organization’s effectiveness while sharing information with customers, suppliers, and regulatory agencies. The authors highlight traceability as a source of competitive advantage. 

In this sense, [40] will develop a study and present its impacts, such as the efficiency and efficacy (Table 2) that a traceability system generates for the actors in the network.

Narsimhalu et al. (2015) [41] discuss (from the distributors’ points of view) the impacts that the deployment of a traceability system can have on safety and quality, in terms of the improvement in supply chain performance, the reduction of the cost of replacement and risk, supply chain sustainability, and effective inventory management.

Canavari et al. (2010) [37] affirm that a traceability system implemented only to answer a legal question should not be seen as a system that creates value for a company. However, a traceability system can create value by exceeding legal standards. In this sense Dabbene et al. (2014) [36] state that a traceability system involves managerial decisions about the value chain that go beyond product substitution in case of crisis and falsification prevention. Supply chain actors typically assign different values to traceability, such as increasing the efficiency of management processes and risk management. For consumers, this represents added value that is mainly related to safety and quality. For producers, this is a tool to avoid a break in the market supply that can strengthen the brand, in addition to ensuring the compliance with required policies.

Alfaro and Rábade (2009) [23] argue that traceability is more than a mechanism to ensure safety: it represents a strategic tool that impacts the management of operations which have an impact on inventory management. However, in the current context of unpredictability and increasing variability, companies need secure and resilient chains to maintain their business. 

The objectives and benefits of a drug traceability system are mainly related to the patients’ safety and quality of life. Additionally, a previous study mentions the optimization of the inventory and the production plan (to minimize losses), and the improvement of accuracy and production time [7]. 

Digital technologies can bring a number of potential benefits in supply chain management. The use of the Internet of Things in supply chains can bring visibility to each item and generate a visible supply chain, allowing the location and characteristics of all the materials and parts in the supply chain to be identified at any point in time. Studies have noted the importance of supply chain visibility in the operational processes and management of the pharmaceutical industry, highlighting that it is one of the most complex and vital sectors in all business fields [42,43,44]. 

Table 3 was elaborated on based on the studies discussed in this article and consolidates the expected values of a traceability system. 

#### 2.2.2. Research Framework Based on the Technology–Organization–Environment (TOE)

**Figure 2:**
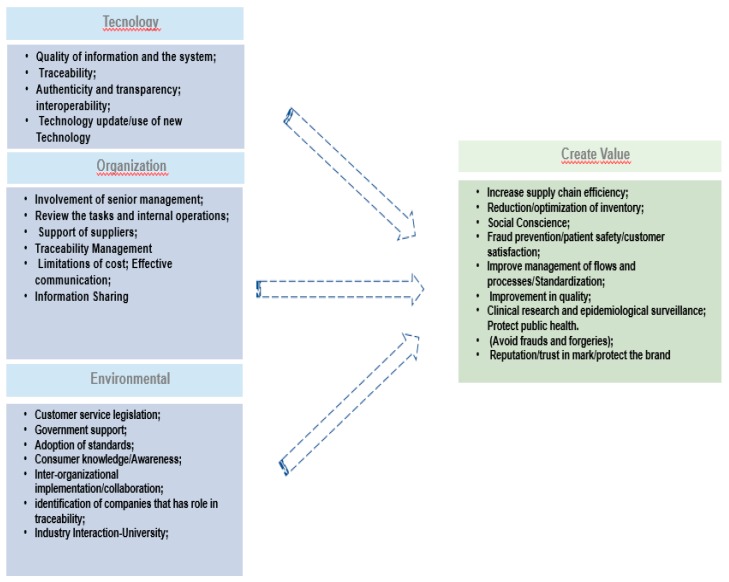
Research framework of the critical success factors for a drug traceability system (adapted from [31]).

Based on the literature review, we obtained a set of CSFs and values, which were defined in the literature as presented in Figure 2. 

Technological factors refer to the characteristics of the technological environment, which can influence the diffusion of the drug traceability systems in an organization. These factors include various dimensions of technology, such as the relative advantage of the quality of information in the system, which increase the diffusion of information technology in the organization. Organizational factors refer to the scope, resources, and size of an organization, and the environmental dimension refers to the context in which the organization develops its industry, competitors, and government relations [45]. TOE is a well-known and widely used framework in technology adoption and diffusion [31]. The TOE framework has been widely used to identify the main drivers of inter-organizational innovation for partners [46,47,48]. 

## 3. Methods

This study obtains qualitative information from PSC experts in order to assess perspectives related to CSF for an anticipated Brazilian national drug traceability system. Qualitative elements were gathered using the following approaches:

A questionnaire issued to PSC experts;

In-depth follow-up interviews with PSC experts to validate CSF based on framework research (Figure 2). The interview script was elaborated based on a theoretical review, and structured based on the CSFs discussed in the literature and the concept of value creation with the adoption of traceability systems. The main objective of the interviews was to understand the value created by the implementation of the drug traceability system and the respective enabling factors for the implementation of a successful drug traceability system. After the validation of the CSF, the AHP method was applied in order to establish a rank for the factors according to the general objective of this research.

### 3.1. Expert Recruitment and Profiling

Interviews were conducted with 17 professionals of different levels (Table 4), with the objective of creating a managerial and operational vision. Interviews were conducted with professionals working in different parts of the drug supply chain (laboratory, distributors, and hospitals). The professionals had an average of more than 15 years of overall experience in their fields and an average of about five years of experience in handling traceability issues. The professionals who participated in the interviews are experts on the topic of drug traceability, are active participants in traceability projects in their companies, and all work in upper management.

### 3.2. Analytic Hierarchy PROCESS (AHP) Approach

The AHP was developed by Thomas L. Saaty in the early 1970s and is a well-known and widely used multicriteria decision-making process to support decision-making in negotiated dispute resolutions. The AHP is based on the comparison of criteria pairs followed by the application of a process to calculate the relative importance of each criterion. Then, using peer comparison, the alternatives are scored against the criteria to determine the best overall candidate. Saaty (1990, 1994) [49,50] defined three main operations for the AHP, namely:

(1) Construction of hierarchies: The problem with AHP is that it is structured in hierarchical levels, as a way of seeking a better understanding and evaluation of the problem being analyzed. The construction of hierarchies is a basic stage of the process of human reasoning. In this study, we identify the key elements of the decision making and group them in similar sets, which are placed in specific layers;
(2) Definition of priorities: Priority setting in AHP is based on human beings’ ability to perceive the relationship between observed objects and situations by comparing pairs in the light of a particular focus or criterion (parity judgments);
(3) Logical consistency: In the AHP, it is possible to evaluate the consistency of the constructed prioritization model. The AHP begins by decomposing the problem into a hierarchy.

The scale recommended by [50], shown in Table 5, ranges from 1 to 9, where 1 means the lack of importance of one criterion to another, and 9 means the extreme importance of one criterion to another, with intermediary stages between 1 and 9. Disregarding the comparisons between the criteria themselves, which represent 1 on the scale, only half of the comparisons need to be made, as the other half consists of the reciprocal comparisons in the comparison matrix, which are the already compared reciprocal values.

After the matched comparisons are performed through the previously created hierarchy and the fundamental table developed by [49], a square matrix is created. In this matrix, by convention, the results of the comparisons between the element in the left column in the row with the element that is displayed in the first row of the comparison column are displayed. Table 6 presents an example of a judgment matrix used in the AHP method. In the example, a total of five criteria are compared to pairs.

The values on the diagonal of the comparison matrix, starting at the comparison between the pair AA, are always equal to one. This is due to the fact that the element has equal importance when compared with itself. The scale presented in Table 1, proposed by [49], is used in order to fill the other comparisons. Judgments are made by comparing the line element to the respective pairs in the columns. Judgments are reciprocal, that is, values are presented inversely to the weight assigned in the evaluation. Therefore, when criterion “A” is seven times more important than criterion “D”, when compared to criterion “A”, criterion “D” will present one-seventh of its strength.

Consistency Index (*CI*) 

Peer judgments must be normalized in order to obtain the Consistency Index (*CI*). According to [51], the *CI* is obtained by the following equation:

*CI* = (*λ* − *n*)/*n* − 1

where *λ* is the largest matrix eigenvector, equivalent to Eigen’s main number, and n is the number of criteria of the matrix

Although the decision is based on the values and preferences of the decision maker, a series of specific criteria or objectives can be used to prioritize the projects and determine the real meaning of the optimal relationship. These criteria must be grouped in sets to be in line with their objectives. 

## 4. Results

To create the AHP questionnaire, the TOE framework was adopted to group the critical factors validated from the literature and other factors discussed by experts. 

As a result of study, we obtained a set of CSFs, which were determined based on the literature review and validated by the professionals who participated in the study, as presented in Table 7. 

The AHP was modeled based on Table 7 to calculate the weights of the criteria and sub-criteria. After the validation of the weights obtained from the application of the method, it was necessary to calculate the consistency ratio (CI) in order to verify the consistency of the responses assigned by the decision maker. The CI found for the responses given by the decision maker was:

CI = 0.039

that is, below the limit of 0.10.

After calculating and collecting the results according to the steps of the AHP method, the products of the matrices with the parity comparisons between the criteria and the sub-criteria were transferred to a spreadsheet, from which Table 8 was generated. 

The authors of [8,30] suggest the use of the TOE model to evaluate the adoption of new and innovative technologies and information systems; they indicate that the main factors that should be considered in the implementation of a traceability system are divided into three categories: organizational, technological, and environmental. In this context, the results of this study were grouped using these categories.

In a first comparative analysis, the most important criteria tend to be those linked to organization (62.4%), followed by those linked to technology (28.7%) and environment (8.9%). There is also a tendency for hospitals to share this view with laboratories. This is a slightly different view from that proposed by [48], who suggest that technological criteria are more relevant for hospitals for the implementation of a system than organizational ones.

### 4.1. Discussion

The TOE (Technology-Organization-Environment) framework can support understanding the critical factors that contribute to successful adoption and implementation of the drug traceability system [30]. 

#### 4.1.1. Technology

In the criteria linked to technology, the quality of information and the system traceability stand out from the other sub-criteria, appearing in second position in the prioritization developed by the specialists aligned to authors [8,22,31,34,37]. In [31], It is reported that users of traceability systems are concerned with issues related to the quality of information and the system, as well as with friendly use. These concerns are based on the correct understanding of the complete, and reliable information needs of users of traceability systems. This is also linked to the preliminary results of the interviews conducted in the present study, in which the participants pointed out that the generation of a well-defined implementation roadmap is an important part of the creation of a high-quality drug traceability system.

In the results related to technology, the fifth position in the prioritization was awarded to transparency, authenticity, and access. This aligned with [33], which claims that the transparency of a supply network is important since all stakeholders have a shared understanding of access to information related to the product and process that they have requested, without loss, noise, delay, or distortion. Transparency enables the stakeholders to achieve efficient chain-level shipments, when it is necessary to rely on anticipated alerts, in the event of a potential emerging problem, through a proactive quality-monitoring system to optimize the supply chain.

#### 4.1.2. Organization

Within the organizational criteria, the involvement of top management was highlighted in the reviewed literature and was concluded by experts in traceability systems to be the main factor affecting the development of a drug traceability system. Management support is vital to ensure that priority is given to the implementation of the traceability system, as discussed by [8,31,37]. In addition, experts mentioned that of a lack of digital skills and attitudes across the board at senior and middle management levels as well as within day-to-day factory operations. 

Within the organizational criteria, data governance and the reviewing of internal tasks and operations stand out among the five main positions pointed out by the specialists. Canavari et al. (2010) [37] claim that a company’s forms of coordination, complexity, and willingness to create long-term relationships with other actors in the drug supply chain strongly affect the opportunities to collect and manage information. However, the exchange of information between actors in a supply chain can enable proactivity for traceability. Furthermore, in [34] it is pointed out that quality and safety of drugs are shared responsibilities of all actors in the chain, including government, industry, and consumers. Regarding the necessary revisions to the internal operations of the drug supply chain, Ngai et al. (2007) [33] warn that organizations, groups, and individuals often resist change. Thus, it is best to avoid major changes in the existing operations, involving staff in the process of change whenever possible. 

#### 4.1.3. Environment

Results related to environment were highlighted by expert in factors such as: legislation compliance, government support, knowledge of the consumer/awareness, industry-university interaction, interorganizational implementation/contribution and adoption of standards. It was surprising that compliance with legislation appeared in a low position on the specialists’ priority list. During the preliminary interviews with the experts, this point was always strongly highlighted and considered to be very relevant. Similarly, in the results, this question has some relevance, since it is placed first among the criteria related to the environment, although in the general context it was placed in twelfth position. Industry–university interaction is a similar point to that of compliance with legislation. Such an interaction has been widely discussed in congresses and symposiums as a relevant point for the implementation of a drug traceability system. The importance of industry–university interaction was highlighted by [33] as a critical factor for the implementation of a drug traceability system, since universities are knowledge-gathering institutions capable of leveraging innovation for industry. However, for Brazilian specialists, this is the smallest of the critical factors. 

CSFs should serve as a foundation for a successful deployment that allows companies to capture values, beyond compliance with legislation. 

#### 4.1.4. Theoretical Implications

The TOE framework is an appropriate and understandable way to group factors that affect the decision-making process for the adoption of innovative IT systems. From a theoretical perspective, the current work identifies the clear characterization of the critical factors of a drug traceability system as a more relevant contribution. Addressing the aforementioned gaps in the literature, this paper contributes to the identification of, and the determination of the relative importance of, each CSF for the implementation of a traceability system in the drug chain. In addition to the factors discussed in the literature, other points were discussed by the experts interviewed in this study and complemented in the list of CSFs as data governance according to Table 8. 

The experts interviewed in this study emphasized that a centralized IT and organizational structure is imperative for data governance. The centralization of IT can play an enabling role in enabling the efficient operation of a drug traceability system, as it reduces the need to allow businesses to enact their own policies or to try to reconcile potentially conflicting rules. The value creation of a drug traceability system introduces the need for a comprehensive and effective data governance policy. Data governance policies should be defined as one of the core guidelines of a high-quality system. Events that result from interactions between a mix of participants in a drug supply system depend on governance mechanisms that may be exceptionally difficult to understand.

The biggest effect that legislation would have on the development of a drug traceability system in Brazil is the creation of a database that is centralized in a federal government institution for the tracking of data storage and consultation regarding drugs under the institution’s responsibility. In this way, the characteristics of the NDS have been changed in order to seek clarity on a variety of subjects (in particular data governance) through the establishment of new rules for traceability. ANVISA was responsible for the creation of a new RDC in order to comply with law no. 13,410/2016, thus creating RDC no. 157/2017. This RDC establishes the mechanisms and procedures for drug tracking.

Additionally, other factors were mentioned as CSFs by the specialists interviewed in this study, such as the standardization of products, registration, team training, the definition of equipment and data networks, the detailed monitoring of the timetable, and drug testing. 

#### 4.1.5. Management Implications

From a practical point of view, the contribution of this research is that it provides experts’ insights on the topic of drug traceability, especially concerning how possible values can be captured by companies and what factors are critical to achieving those values. Another contribution of this work is that it presents comparisons of the CSFs found in the literature with the opinions of experts through the application of AHP. Traceability is also a key element for continuous improvement. Through a system that allows information about drugs to be traced, a company can identify opportunities to improve processes, products, and services.

## 5. Conclusions

In line with the questions that guided this study, the present analysis identified the critical success factors for the implementation of a drug traceability system based on the views of several professionals who are directly involved with the implementation of drug traceability projects. Through their systematic participation, these professionals, who are specialists in the field of pharmaceutical research, discussed the research questions proposed in the present study, sharing opinions, including opinions of judgment. This process allowed for the enrichment of the proposed ideas, and also facilitated the discussion of more controversial subjects. 

There are several limiting factors for the development of a drug traceability system in Brazil. For example, the drug traceability scenario in Brazil is not consolidated. The study of drug traceability systems in Brazil is at an early stage, and will be the subject of many other discussions.

The scenario analyzed in the present study, in which there are various interests among the actors, also opens the opportunity for future academic research. The results show that data governance is a topic of extreme importance and merits more study. It is necessary to have a data governance structure that works according to data management. Data governance has recently evolved to formally provide clarity in policies and standards, data quality measurements, roles and responsibilities, and changes that allow the value of information assets to be maximized. Data governance is important due to the need for lengthy discussions that result in the amendment of the law regarding drug traceability.

## Figures and Tables

**Table 1:** Critical success factors (CSFs) for the development of a drug traceability system.

Critical Success Factors	Authors							
	Ngai et al. (2007) [33]	Alfaro e Rábade (2009) [23]	Canavari et al. (2010) [37]	Miao et al. (2011) [8]	Aung e Chang (2014) [34]	Rotunno et al. (2014) [4]	Duan, et al. (2017) [31]	Theyel (2017) [7]
Legislation	√		√	√	√		√	
Government Support				√			√	
Effective communication		√			√	√		
Top management involvement	√		√	√			√	
Supplier Support	√	√		√			√	
Consumer Knowledge				√			√	
Quality of information and traceability system		√	√	√	√	√	√	
Interação Indústria – Universidade	√							
Interoperabilidade					√			
Traceability data management	√		√		√			
Transparency, authenticity and access					√			
Sharing Information					√	√		
Technological Update/Use of new technologies.	√		√					√
Inter-organizational implementation/Collaboration	√			√	√	√	√	
Adoption of Standards								
Review of tasks and internal operations	√							√
Identification of companies that play a role in traceability								
Cost	√		√					

**Table 2:** Impacts of a drug traceability system.

Impact on Efficiency-Company’s Ability to Manage Its Operating Costs	Impact on Effectiveness-Ability to Affect the Market in Which It Operates
Recall/Withdrawal expenses	Brand Protection
Inventory Costs	Product Quality Management
Acquisition Cost	Reputation Improvement
Sales Costs	Product differentiation
	Notify stakeholders

Source: Author, adapted from [40].

**Table 3:** Value creation of a drug traceability system.

Expected Values	Lovis (2008) [39]	Alfaro & Rábade (2009) [23]	Canavari et al. (2010) [37]	Dabbene, Gay e Tortia (2014) [36]	Epelbaum e Martinez (2014) [40]	Narsimhalu, Potdar e Kaur (2015) [41]	Theyel (2017) [7]
Increased Supply Chain Efficiency	√				√	√	
Preventing errors/Reliability/improves accuracy	√	√			√	√	√
Protect public health. (Avoid Fraud and Falsification)	√				√		√
Allow medical-legal investigations	√						
Clinical research and epidemiological surveillance	√						
Improve flow and process management	√	√	√	√	√		√
Increased Profit/Cost/Loss Reduction/Inventory Optimization		√				√	√
Employee Satisfaction/Shared Value			√				√
Fraud Prevention Measures/Patient Safety/	√			√			
Improvement in quality				√	√	√	
Reputation/Brand					√		
Innovation							√
Flexibility							
Differentiation of products/competitive advantage		√			√		

**Table 4:** Profiles of questionnaire respondents. ANVISA—National Agency of Sanitary Vigilance

Actor of the Pharmaceutical Supply Chain (PSC)	Organization	Occupation
Governmental agency	Ministry of Health	1. Director of the Industrial Complex and Innovation in Health
	ANVISA	TI Manager of ANVISA
Laboratories	Laboratory-A (LA)	2. Pharmaceutical Coordinator of Production; 3. Logistics Manager: 4. Logistics Coordinator/Person in charge of Factory Shipment: 5. Systems Coordinator:
	Laboratory-B (LB)	6. Senior Suppliment Manager 7. Senior Projects Manager-Serialization and Traceability Project
	Laboratory-C (LC)	8. IT Manager
Hospitals	Hospital-A (HA)	9. Infrastructure and Logistics Director: 10. Logistics Manager: 11. Manager of Innovation Projects - Center for Technological Innovation of the Hospital: 12. Logistics Person in charge of receiving at the Hospital's Central Supply.
	Hospital-B (HB)	13. Corporative Coordinator
Distributor/Distribution Center	Distributor-A (DA)	14. Logistics Operator Responsible for the Reception, Separation, and Expedition at the Manufacturer’s Distribution Center: 15. Logistics Person Responsible for the Reception, Separation, and Expedition at the Hospital’s Distribution Center.
Association	Brazilian Association of Pharmacy and Drugstore Networks	16. Presidential Advisor

**Table 5:** Basic Analytic Hierarchy Process (AHP) scale of Saaty.

Intensity of Importance	Definition	Explanation
1	Equal importance	Both criteria contribute equally to the objective.
3	Moderate importance	The evaluator’s experience says that one element has little greater importance than the other for the objective
5	Strong importance	The evaluator’s experience says that one element has greater importance than the other for the objective
7	Very strong importance	The evaluator’s experience says that one element has relatively greater importance than the other for the objective
9	Extremely important	The evaluator’s experience says with a high degree of certainty that one element is of greater importance than the other in relation to the objective
2, 4, 6, 8	Intermediate values	Used when an intermediate index of importance is required

**Table 6:** AHP comparison matrix with five criteria.

	A	B	C	D
A	1	2	3	7
B	1/2	1	2	4
C	1/3	1/2	1	3
D	1/7	1/4	1/3	1

**Table 7:** CSFs for the implementation of a drug traceability system.

DIMENSION	FACTORS
Technology	Quality of Information and Traceability System
	Interoperability
	Transparency, authenticity, and access
	Technological update/Use of new technologies
Organization	Involvement with senior management
	Supplier support
	Data governance
	Traceability Management
	Effective communication (training-divulgation)
	Sharing Information
	Revision of internal tasks and operations
	Cost limitations
Environment	Legislation compliance
	Government support
	Knowledge of the consumer/awareness
	Industry-University interaction
	Interorganizacional implementation/Contribution
	Adoption of Standards
	Identification of the companies that have a role in traceability

**Table 8:** Prioritization of the CSFs.

Criteria	Sub-Criteria	Average	Prioritization
Organization	Involvement with senior management	21.0%	1
Technology	Quality of Information and Traceability System Management	13.1%	2
Organization	Data governance	9.8%	3
Organization	Revision of internal tasks and operations	8.6%	4
Technology	Transparency, authenticity, and access	6.6%	5
Technology	Interoperability	6.5%	6
Organization	Supplier support	6.3%	7
Organization	Cost limitations	6.0%	8
Organization	Effective communication (training-divulgation)	5.5%	9
Organization	Sharing Information	5.2%	10
Technology	Technological update/Use of new technologies	2.5%	11
Environment	Legislation compliance	2.5%	12
Environment	Government support	1.6%	13
Environment	Adoption of Standards	1.3%	14
Environment	Knowledge of the consumer/awareness	1.1%	15
Environment	Interorganizacional implementation/Contribution	1.0%	16
Environment	Identification of the companies that have a role in traceability	0.8%	17
Environment	Industry-University interaction	0.5%	18
		100%	
